# Identifying patients at highest-risk: the best timing to apply a readmission predictive model

**DOI:** 10.1186/s12911-019-0836-6

**Published:** 2019-06-26

**Authors:** Natalie Flaks-Manov, Maxim Topaz, Moshe Hoshen, Ran D. Balicer, Efrat Shadmi

**Affiliations:** 1 Clalit Research Institute, Clalit Health Services, Shoham 2, Ramat Gan, Israel; 2 Cheryl Spencer Department of Nursing, Faculty of Social Welfare and Health Sciences, University of Haifa, 31905 Haifa, Israel; 3 Department of Epidemiology, Faculty of Health Sciences, Ben-Gurion University of the Negev, Beer-Sheva, Israel

**Keywords:** Timing of readmission predictive model, All-cause readmission, Electronic medical records

## Abstract

**Background:**

Most of readmission prediction models are implemented at the time of patient discharge. However, interventions which include an early in-hospital component are critical in reducing readmissions and improving patient outcomes. Thus, at-discharge high-risk identification may be too late for effective intervention. Nonetheless, the tradeoff between early versus at-discharge prediction and the optimal timing of the risk prediction model application remains to be determined. We examined a high-risk patient selection process with readmission prediction models using data available at two time points: at admission and at the time of hospital discharge.

**Methods:**

An historical prospective study of hospitalized adults (≥65 years) discharged alive from internal medicine units in Clalit’s (the largest integrated payer-provider health fund in Israel) general hospitals in 2015. The outcome was all-cause 30-day emergency readmissions to any internal medicine ward at any hospital. We used the previously validated Preadmission Readmission Detection Model (PREADM) and developed a new model incorporating PREADM with hospital data (PREADM-H). We compared the percentage of overlap between the models and calculated the positive predictive value (PPV) for the subgroups identified by each model separately and by both models.

**Results:**

The final cohort included 35,156 index hospital admissions. The PREADM-H model included 17 variables with a C-statistic of 0.68 (95% CI: 0.67–0.70) and PPV of 43.0% in the highest-risk categories. Of patients categorized by the PREADM-H in the highest-risk decile, 78% were classified similarly by the PREADM. The 22% (*n* = 229) classified by the PREADM-H at the highest decile, but not by the PREADM, had a PPV of 37%. Conversely, those classified by the PREADM into the highest decile but not by the PREADM-H (*n* = 218) had a PPV of 31%.

**Conclusions:**

The timing of readmission risk prediction makes a difference in terms of the population identified at each prediction time point – at-admission or at-discharge. Our findings suggest that readmission risk identification should incorporate a two time-point approach in which preadmission data is used to identify high-risk patients as early as possible during the index admission and an “all-hospital” model is applied at discharge to identify those that incur risk during the hospital stay.

## Background

Interventions that are aimed at the prevention of hospital readmissions are increasingly guided by computerized risk prediction models, which identify high-risk patients [1]. To date, most readmission prediction models are implemented upon patient discharge [2]. A growing body of evidence, however, indicates that interventions that include an early in-hospital component, such as comprehensive discharge planning [3], are key to reducing readmissions, thus, highlighting the need for early, within hospitalization high-risk prediction.

Early high-risk patient identification is becoming increasingly possible. With the advent of electronic health records (EHRs) [4], detailed data on key risk factors, including clinical and healthcare utilization, are also available from the preadmission period [5]. Previously, we showed that such a pre-admission prediction model (the Preadmission Readmission Detection Model [PREADM]) provides accurate high-risk assessment [6]. Similarly, a multi-condition electronic model, based on data available at admission, showed that meaningful patient-level risk stratification of readmission risk can occur early in the hospital stay without the need to wait for further information at time of discharge [7]. A recent review has demonstrated that preadmission prediction models performed comparably well to the at-discharge models [2].

Whether identification of patients at high-risk for readmission should be performed at the beginning or at the end of the index hospitalization is not only a question of predictive accuracy, it also depends on the types of readmission prevention interventions to which high-risk patients are referred. Thus, in-hospital interventions can benefit from early high-risk identification of targeted patients, and programs targeting the post-hospitalization phase, should rely on risk prediction at the time of discharge. Thus, the trade-off between early versus at-discharge prediction and the optimal timing of high-risk case identification remains to be determined. To address this gap, the aim of this study was to examine a high-risk patient selection with readmission prediction models using data available at two time points: (1) at admission and (2) at the time of hospital discharge.

## Methods

### Study design and setting

We conducted a historical prospective cohort study of adult members from Clalit Health Services (Clalit), the largest of four integrated payer-provider health funds, which covers over 52% of the Israeli population (more than 4.2 million patients). Clalit’s data warehouse includes clinical information, administrative data on patient demographics and healthcare service utilization, community clinic information (preventive care, risk factors, primary care and specialist visits), hospital records, and laboratory and pharmacy data (prescribing and dispensing).

### Study population

Our cohort included all hospitalized older adults (≥65 years) discharged alive from internal medicine units in one of Clalit’s eight general hospitals in 2015 (1/1/2015 to 31/12/2015). We excluded individuals who died during the index hospitalization, were transferred to another facility, or did not have continuous membership in Clalit 1 year before the index hospitalization and 30 days after (less than 1% of the Clalit membership). Hospitalizations with lengths of stay (LOS) of less than one night were also excluded to avoid including observation stays. All datasets were made anonymous, in keeping with the standard operating procedures of Clalit’s Data Extraction Committee. The study was approved by Clalit’s institutional review board.

### Study outcome

The outcome of interest was all-cause 30-day emergency (unplanned) readmissions to any internal medicine ward at any hospital in Israel.

### Study predictors

To compare the preadmission with at-discharge prediction models, we used the previously validated PREADM model and developed a new model incorporating PREADM [6] with hospital data (PREADM-H). For the at-discharge model we used combined preadmission and within-hospitalization data, as this approach has previously shown the highest prediction accuracy [8].

The PREADM allows early identification of high-risk patients upon hospital admission to an internal medicine unit [6]. The PREADM has been in use in Clalit since 2012 to direct the readmission prevention strategy for high-risk patients on the second day of admission to any hospital throughout Israel, and in primary care interventions aimed at counseling high-risk patients upon discharge from the hospital. The model includes: six chronic conditions (congestive heart failure, chronic obstructive pulmonary disease, chronic renal failure, malignancy, arrhythmia, and disability), number of primary care and specialist visits, number of days since last hospitalization, number of hospital admissions in the past year, body mass index, and an indicator for the hospital’s catchment area.

Data from the index admission period were based on variables from the widely used and well-validated HOSPITAL model [9]. The HOSPITAL model was previously incorporated into an admission model showing good discriminatory power (C-statistic of 0.72 in the United States and Canadian hospitals and 0.68 in Swiss hospitals) [10].

The risk factors included the above 11 variables from the PREADM and six unique predictor variables (not including the number of previous hospitalizations, as it already appears in the PREADM) from the HOSPITAL model, including last available hemoglobin before discharge, discharge from oncology treatment, last available sodium level before discharge, any procedure performed during the index admission, type of index admission, and LOS.

### Data analysis

We compared the characteristics of patients with and without 30-day readmission using the chi-squared tests for categorical variables and t-tests for continuous variables. For derivation of the combined PREADM-H model we randomly split the sample into separate derivation (70%) and validation cohorts (30%). We used the generalized estimating equations approach, as admissions are nested within individuals [11]. We assessed the model’s discrimination using the validation cohort with the C-statistic that measures the trade-off between true positives and false negatives at all possible thresholds. Model calibration was assessed by comparing predicted with observed probabilities of readmission by top decile and quintile of risk. For each model (PREADM and PREADM-H), we calculated the positive predictive value (PPV) for the 10 and 20% highest risk categories.

We then compared the percentage of overlap between the models for each of the 10 and 20% cut-points (i.e., the percentage of patients identified as being in the same high-risk category by each model separately and by both models) and calculated the PPV for the subgroups identified by each model separately and by both models. We conducted all analyses using R version 3.2.2.

## Results

**Fig. 1:**
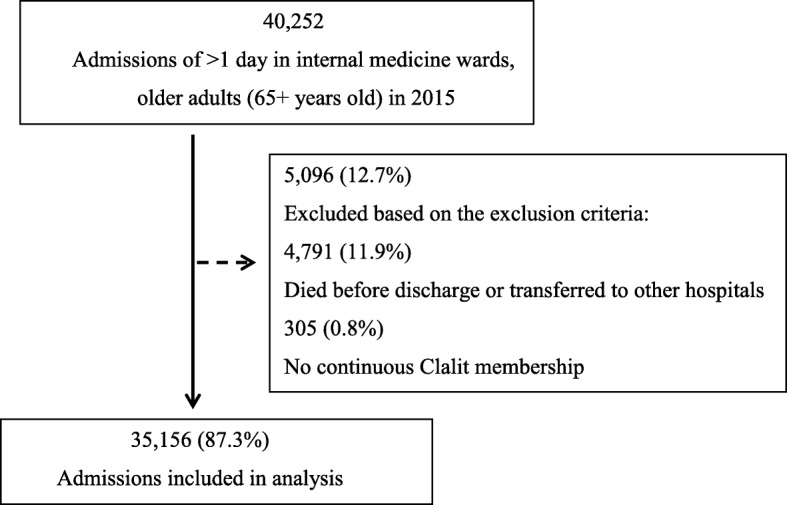
Flowchart of the Study Population

The final cohort included 35,156 index hospital admissions (24,510 unique inpatients) after we excluded 5096 patient’s admissions who died before discharge or did not have a continuous membership in Clalit. The flow-chart for selection of the study's population appears in Fig. 1. The study population was 47.9% male, 78.9 years of age on average, and predominantly Jewish (88.4%). The mean index hospital admission lasted 5.3 days, and 6933 (19.7%) index admissions resulted in 30-day readmission (Table 1). Patients who were readmitted differed from non-readmitted patients in terms of their demographic, clinical and prior health service use characteristics.

**Table 1:** Baseline cohort characteristics of 35,156 index admissions

Characteristics	Index admission *N* = 35,156	No readmission *N* = 28,223 (80.3%)	Readmission *N* = 6933 (19.7%)	*P* value
Age, y, mean (SD)	78.9 (8.2)	78.8 (8.2)	79.3 (8.1)	< 0.001
Male, *n* (%)	16,855 (47.9%)	13,413 (47.5%)	3442 (49.6%)	0.002
Socioeconomic status, *n* (%)				
Low	7135 (20.6%)	5470 (19.7%)	1665 (24.3%)	< 0.001
Medium	15,270 (44.0%)	12,288 (44.1%)	2982 (43.5%)	
High	12,288 (35.4%)	10,075 (36.2%)	2213 (32.3%)	
Ethnicity, *n* (%)				
Jewish	31,083 (88.4%)	25,144 (89.1%)	5939 (85.7%)	< 0.001
Arabs	4073 (11.6%)	3079 (10.9%)	994 (14.3%)	
Before index admission				
CHF, *n* (%)	10,964 (31.2%)	8175 (29.0%)	2789 (40.2%)	< 0.001
COPD, *n* (%)	7879 (22.4%)	5957 (21.1%)	1922 (27.7%)	< 0.001
CRF, *n* (%)	11,314 (32.2%)	8513 (30.2%)	2801 (40.4%)	< 0.001
Malignancy, *n* (%)	10,486 (29.8%)	8163 (28.9%)	2323 (33.5%)	< 0.001
Arrhythmia, *n* (%)	14,842 (42.2%)	11,551 (40.9%)	3291 (47.5%)	< 0.001
Disability, *n* (%)	11,742 (33.4%)	8680 (30.8%)	3062 (44.2%)	< 0.001
Oncology (treatment phase), *n* (%)	6064 (17.2%)	4648 (16.5%)	1416 (20.4%)	< 0.001
Body mass index^a^, mean (SD)	28.2 (6.1)	28.3 (6.0)	27.9 (6.3)	< 0.001
No. hospital admissions in the past year, mean (SD)	1.6 (2.2)	1.3 (1.9)	2.6 (2.9)	< 0.001
No. primary care and specialist visits in the past year, mean (SD)	16.6 (13.4)	16.3 (13.0)	17.6 (15.0)	< 0.001
Residing in hospitals’ catchment area, *n* (%)	65–4342 (0.2–12.4)	51–3541 (0.2–12.5)	14–982 (0.2–13.4)	< 0.001
No. days from last hospitalization**,** mean (SD)	205 (150)	219 (148)	147 (144.6)	< 0.001
During index admission				
Index admission LOS, days, mean (SD)	5.3 (5.7)	5.2 (5.7)	5.9 (5.8)	< 0.001
Procedure, *n* (%)	1956 (5.6%)	1645 (5.8%)	311 (4.5%)	< 0.001
Index admission type: urgent, *n* (%)	34,119 (97.1%)	27,312 (96.8%)	6807 (98.2%)	< 0.001
Hemoglobin level^b^ (last) < 12 g/dL, *n* (%)	20,355 (58.0%)	15,607 (55.5%)	4748 (68.6%)	< 0.001
Sodium level^c^ (last) < 135 mEq/L, *n* (%)	5187 (14.8%)	3912 (13.9%)	1275 (18.4%)	< 0.001

*Abbreviations*: *y* years, *SD* Standard deviation, *CHF* Congestive heart failure, *COPD* Chronic obstructive pulmonary disease, *CRF* Chronic renal failure, *No* Number, *LOS* Length of stay, *Procedure* any ICD-9 coded procedure, such cardiac catheterization, or diagnostic radiology

^a^Missing values contributed to 0.8%

^b^Missing values contributed to 0.3%

^c^Missing values contributed to 0.2%

Model derivation was performed on 24,599 admissions and tested on 10,557 admissions. Our final model included 17 variables; the 11 PREADM model variables, and six from the HOSPITAL model. In the validation cohort, the PREADM-H model had fair discrimination, with a C-statistic of 0.68 (95% CI: 0.67–0.70). The PPV of the PREADM-H model in the highest risk categories (top 10 and 20%) was 43.0 and 36.1%, respectively and sensitivity and specificity in top 10% was 21.1 and 92.9% respectively (Table 2).

**Table 2:** Prediction of 30-day readmission based on PREADM-H model variables (Derivation cohort, *N* = 24,599)

Variables	OR	(95% CI)	*P* value
Chronic condition			
CHF^P^	1.16	(1.07–1.25)	< 0.001
COPD^P^	1.19	(1.10–1.29)	< 0.001
CRF^P^	1.18	(1.09–1.27)	< 0.001
Malignancy^P^	1.02	(0.93–1.13)	0.658
Arrhythmia^P^	1.03	(0.96–1.11)	0.446
Disability^P^	1.30	(1.21–1.40)	< 0.001
Oncology (treatment phase)^H^	1.13	(1.00–1.26)	0.041
Body mass index^P^	0.99	(0.98–0.99)	< 0.001
No. hospital admissions in the past year^P^	1.13	(1.10–1.15)	< 0.001
No. primary care and specialist visits in the past year^P^	1.00	(1.00–1.00)	0.522
Residing in hospital’s catchment area^P^	0.65–1.62		0.06–1.00
No. days from last hospitalization^PH^	1.00	(1.00–1.00)	< 0.001
Index admission LOS^H^ > =5 d	1.26	(1.18–1.35)	< 0.001
Procedure (any ICD-9 codes) during hospital stay^H^	0.84	(0.71–0.98)	0.026
Index admission type^H^: urgent	2.09	(1.65–2.66)	< 0.001
Low hemoglobin level at discharge^H^ (< 12 g/dL)	1.28	(1.19–1.38)	< 0.001
Low sodium level at discharge^H^ (< 13 mEq/L)	1.26	(1.15–1.38)	< 0.001
Model Performance	(top 10%)	(top 20%)	
PPV	43.0 (40.0–46.0)	36.1 (34.1–38.1)	
Sensitivity	21.1 (19.4–22.9)	37.5 (35.4–39.6)	
Specificity	92.9 (92.4–93.5)	84.5 (83.7–85.3)	
C-stat (validation cohort, *n* = 10,557)	0.68 (95% CI: 0.67–0.70)		

*Abbreviations OR* Odds ratio, *CI* Confidence interval, *CHF* Congestive heart failure, *COPD* Chronic obstructive pulmonary disease, *CRF* Chronic renal failure, *LOS* Length of stay, *C-stat* Model’s discrimination and calibration, *PPV* Positive predictive value

P: variables from PREADM model

H: variables from HOSPITAL model

PH: variables from PREADM-H model

PREADM-H: Preadmission Readmission Detection Model + Hospital model

**Fig. 2:**
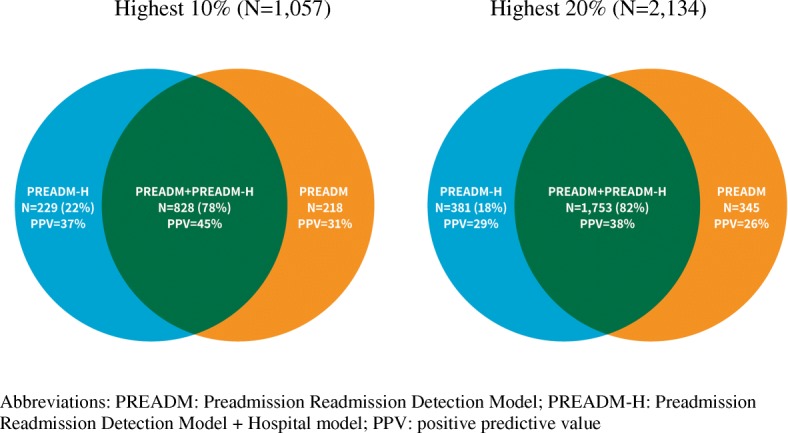
Model Performance for Different Cutoff Points of the PREADM-H vs. PREADM model

**Fig. 3:**
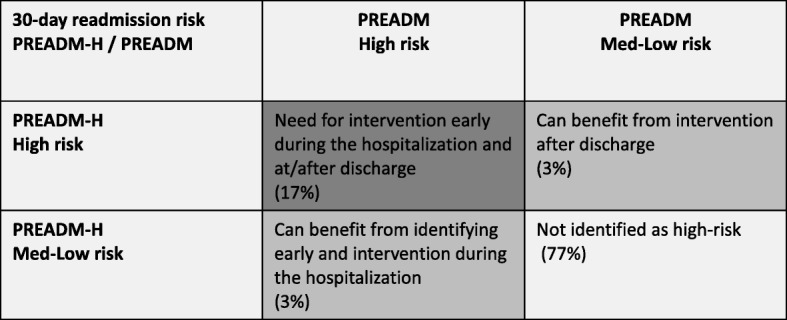
Patient’s detected at high risk for 30-day readmission at-admission (PREADM) vs. at-discharge (PREADM-H) (*N* = 35,156)

Figure 2 shows that 78% of those categorized by the PREADM-H at the highest decile of risk (with a PPV of 45%) were classified similarly by the PREADM model. The remaining 22% who were classified by the PREADM-H highest decile, but not by the PREADM, had a PPV of 37%. Conversely, those classified by the PREADM into the highest decile but not by the PREADM-H (*n* = 218) had a PPV of 31%. A similar picture emerged when examining the differences in populations identified as the 20% highest risk group. In the highest quintile, 82% of those categorized by the PREADM-H (with a PPV of 38%) were also categorized at the highest quintile by the PREADM. The PPV for the remaining 18% who were not identified by the PREADM at the highest quantile was 29%. Thus, applying the PREADM at baseline and PREADM-H at-discharge allowed for accurate detection of an additional 85 subsequently readmitted patients (37% of 229 patients), with a cutoff point for the 10% highest risk group, and 110 patients (31% of 359 patients), using a cutoff point for the 20% highest risk group, who would have otherwise been missed. A detailed account of percent of patients detected at high-risk for 30-day readmission at-admission (PREADM) vs. at-discharge (PREADM-H) appears in Fig. 3.

The characteristics of the two non-overlapping populations appear in Table 3. Patients with a high-risk PREADM score who were not identified as high-risk according to the PREDM-H model had more disability (71.6% vs. 55.5%, *p* value = 0.001), more chronic renal failure (65.1% vs. 50.7%, p value = 0.003), more hospital admissions in the past year (3.9 vs. 3.4, p value = 0.003) and less days from the previous hospitalization (39.2 vs. 54.0, p value = 0.001). Patients who were identified as non-high-risk by the PREADM model but were identified as high-risk by the PREADM-H model have longer LOS (87.3% vs. 15.6% with a LOS of at least 5 days, *p* value< 0.001), a larger percent were in an oncology treatment phase (31.4% vs. 12.8%, *p* value< 0.001), all had an urgent index admission (100% vs. 89.4%, *p* value< 0.001), almost all had a low hemoglobin level (98.7% vs. 49.1%, *p* value< 0.001) and about half had low sodium levels (48.9% vs. 4.6%, *p* value< 0.001).

**Table 3:** Characteristics of the highest risk groups (top 10%), according to each model separately (PREADM-H vs. PREADM)

Characteristics	Total	PREADM-H high risk; PREADM non high risk	PREADM-H non high risk; PREADM high risk	*P* value
	*N* = 1057	*n* = 229	*n* = 218	
Age, y, mean (SD)	78.9 (7.8)	79.2 (7.6)	80.0 (8.3)	0.272
Male, *n* (%)	529 (50.0%)	112 (48.9%)	129 (59.2%)	0.037
Socioeconomic status, *n* (%)				
Low	306 (29.3%)	56 (24.8%)	46 (21.3%)	
Medium	446 (42.6%)	95 (42.0%)	104 (48.1%)	
High	294 (28.1%)	75 (33.2%)	66 (30.6%)	0.420
Ethnicity, *n* (%)				
Jewish	861 (81.5%)	205 (89.5%)	200 (91.7%)	
Arab	196 (18.5%)	24 (10.5%)	28 (12.8%)	0.518
Before index admission				
CHF, *n* (%)	649 (61.4%)	113 (49.3%)	128 (58.7%)	0.059
COPD, *n* (%)	458 (43.3%)	74 (32.3%)	88 (40.4%)	0.095
CRF, *n* (%)	645 (61.0%)	116 (50.7%)	142 (65.1%)	0.003
Malignancy, *n* (%)	438 (41.4%)	89 (38.9%)	85 (39.0%)	1.000
Arrhythmia, *n* (%)	620 (58.7%)	129 (56.3%)	130 (59.6%)	0.541
Disability, *n* (%)	752 (71.1%)	127 (55.5%)	156 (71.6%)	0.001
Oncology (treatment phase), *n* (%)	290 (27.4%)	72 (31.4%)	28 (12.8%)	< 0.001
Body mass index, mean (SD)	27.1 (6.2)	27.8 (7.1)	27.1 (5.1)	0.187
No. hospital admissions in the past year, mean (SD)	5.7 (3.3)	3.4 (1.7)	3.9 (1.8)	0.003
No. primary care and specialist visits in the past year, mean (SD)	20.9 (16.2)	18.6 (14.6)	16.9 (14.2)	0.213
No. days from last hospitalization, mean (SD)	39.3 (39.4)	54.0 (55.0)	39.2 (33.4)	0.001
Residing in hospitals’ catchment area, *n* (%)	1–190 (0.1–18%)	1–48 (0.4–21%)	0–45 (0–21%)	0.696
During index admission				
Index admission LOS > =5 days	622 (58.8%)	200 (87.3%)	34 (15.6%)	< 0.001
Procedure, *n* (%)	26 (2.5%)	8 (3.5%)	15 (6.9%)	0.160
Index admission type: urgent, *n* (%)	1054 (99.7%)	229 (100.0%)	195 (89.4%)	< 0.001
Hemoglobin level (last) < 12 g/dL, *n* (%)	933 (88.3%)	226 (98.7%)	107 (49.1%)	< 0.001
Sodium level (last) < 135 mEq/L, *n* (%)	304 (28.8%)	112 (48.9%)	10 (4.6%)	< 0.001

*Abbreviations*: *y* years, *SD* Standard deviation, *CHF* Congestive heart failure, *COPD* Chronic obstructive pulmonary disease, *CRF* Chronic renal failure, *No* Number, *LOS* Length of stay, *PREADM* Preadmission Readmission Detection Model, *PREADM-H* Preadmission Readmission Detection Model + Hospital model

## Discussion

Our results show that the timing of hospital readmission risk prediction both at admission and discharge should be considered when making the decision regarding which population should and can be identified for inclusion in readmission prevention programs. Use of the PREADM model allowed for early identification of high-risk patients, yet missed a portion (18–22%, depending on whether a 10% or 20% highest risk cut-off was used) whose readmission risk was almost as high. Alternatively, the PREADM-H enabled accounting for risk factors that accrued during the hospital stay, though missed some patients who had an a priori high-risk according to the PREADM and whose actual re-hospitalization rate was much higher than the general population (31% readmission rate). Also, as expected, the clinical characteristics of the population that was identified as high-risk by the PREADM-H model was different than those who were identified by the PREADM model (especially as to the within hospital risk-factors). Malignancy, arrhythmia, and number of primary care and specialist visits in the past year were statistically significantly associated with readmission in the univariate analysis (Table 1), and in PREADM model [6]. Yet, when included in a model with variables from the admission period (PREADM-H) they are no longer statistically significant. This is probably due to the inclusion of the LOS variable, which possibly also indirectly captures the complexity and severity of the patient’s overall condition. This is similar to at least part of the contribution of the above stated variables, which may explain why they were no longer statistically significantly associated with the readmission outcome. Taken together, our findings suggest that readmission risk identification should incorporate a two-time-point approach in which preadmission data are used to identify high-risk patients as early as possible during the index admission (with a PREADM type of algorithm) and an at-discharge “all-hospital” model (such as the PREADM-H model), which is applied to identify those who incur risk during the hospital stay.

A two-time-point risk identification approach is also compatible with evidence that reports on the effectiveness of readmission prevention interventions. Systematic reviews [3, 12, 13] have repeatedly shown that no intervention implemented alone is associated with reduced risk for 30-day readmissions. Rather, interventions including components that are implemented before and after discharge, such as transitional nurse visits and discharge follow-up appointments, achieve the greatest reduction in hospital readmissions [14–17].

While the PREADM model, already in use in Clalit for early identification of high-risk patients and guiding physicians and nurses in prioritizing patients for inclusion in early readmission preventive programs that are tailored to meet their ongoing care needs (e.g., discharge planning or referral to a transitional nurse care). This study shows that there is value in the PREADM-H model being incorporated into practice to inform interventions implemented at the point of discharge, as well as communicated to the primary or ambulatory care teams to allow for selection of patients for inclusion in post-discharge targeted interventions. Our results provide an example of the potential complementary implementation of the predictive models to maximize their power in identifying various groups of high-risk patients for inclusion in within as well as post-discharge interventions.

This study’s findings add to the recent literature that addresses the need for new modeling approaches that provide innovative, actionable insights for risk stratification to improve the ability to prevent hospital readmissions [18]. An example of such an approach is multi-hypotheses causal analysis, which generates meaningful insights from health care claims data, guiding the design of care and intervention programs by developing more personalized interventions based on readmission risk associated with specific comorbidities [18] or improved understanding of causes of asthma-related readmission [19]. This is similar to our approach which identifies various groups of high-risk patients for inclusion in interventions by checking the patient’s risk at different time points throughout the hospitalization, rather than providing broad-scope interventions for all individuals.

Another consideration in the applicability of risk-prediction models relates to the compatibility between the type of analytical approach used for the development of the model and the purpose of the use of the model. For example, a model developed for risk adjustment purposes to allow for fair comparisons amongst hospitals’ readmission rates should be developed retrospectively, for which timely data availability does not play a factor [20]. Yet, a predictive model that evaluates patients at-risk for deciding on inclusion in preventive interventions requires the inclusion of data available in real-time from an EHR [21].

### Limitations

Although the patient sample was taken from a large integrated health fund, and the types of data used are similar to those used by other healthcare systems, our results may not be generalizable to other settings where clinic and hospital data are not linked. Specifically, the type of data available at Clalit’s EHR data warehouse may not be available elsewhere. However, with the growing use of EHRs [4], the data included in the final PREADM-H may be increasingly available to many healthcare organizations.

As to model performance, with a PPV of 43% for a 10% threshold and the C-statistic of 0.68, our model presents fair to good accuracy. Whereas sensitivity and specificity values are very similar to the PREADM model (22.2 and 92.2% in PREADM vs. 21.1 and 92.9% in PREADM-H respectively), the PPV was better than the PREADM model (43% vs. 34.3%) [6]. Nonetheless, our detection accuracy is similar to most current models, with a c-statistic mostly around the 0.7 range [1, 2]. Also, while it is potentially possible to improve the performance of the model, the goal of this study was not to develop a completely new model, but to show how the combination of two validated models presents a comprehensive approach to readmission risk detection. Future studies that may be able to improve model accuracy, should, in addition to model performance take into consideration applicability of models, as tested and reported here.

Another limitation is that our model did not include predictors of readmission that are included in other at-discharge models (e.g., indications of complications, pathology reports, or lab values) [22]. Nonetheless, we used variables from the widely used and validated HOSPITAL model with an aim to increase generalizability and applicability to other healthcare systems. Finally, although our results show that the PREADM-H can be applied at two points, immediately at admission and discharge, this process depends on each hospital’s ability and willingness to operate identification and intervention schemes.

## Conclusions

The timing of readmission risk prediction makes a difference in terms of the population identified at each prediction time point - at admission or discharge. As our model performance data shows, early risk identification (by the PREADM) allowed for accurate detection of patients who were highly likely to be readmitted (actual readmission rate of 31%) but were no longer detected at the highest-risk decile when at-discharge identification was applied (by the PREADM-H). Similarly, application of only early at-admission high-risk detection (PREADM) can miss patients with as high as about 37% likelihood for readmission who are newly identified at-discharge (PREADM-H). Thus, implementing at-admission models allows early identification and intervention but misses a portion of patients who are at high-risk of readmission due to factors accrued during the hospitalization period. Conversely, an at-discharge prediction model not only misses the opportunity for early intervention, but also fails to account for patients at high-risk for readmission who can be identified early in their hospital stay. To account for patient risk as well as the opportunity to intervene in order to address this risk, we recommend a combined and complementary approach of applying readmission risk detection at two-time-points, both at-admission and at-discharge.

## Data Availability

The datasets analyzed during the current study are not publicly available due to public availability would compromise patient confidentiality. The data restrictions are imposed by the Clalit Health Services Data Utilization Committee and the Clalit Health Services Internal Review board in order to protect patient confidentiality. But the anonymous datasets are available from the corresponding author on reasonable request.

## Abbreviations

EHR: Electronic health records
LOS: Lengths of stay
PREADM: Preadmission Readmission Detection Model
PREADM-H: Preadmission Readmission Detection Model with hospital data
